# Editorial: Digital information for patient education, volume II

**DOI:** 10.3389/fpubh.2026.1777890

**Published:** 2026-01-29

**Authors:** Xiaofei Zhang, Feng Guo, Paul Lee

**Affiliations:** 1 School of Management, Harbin Institute of Technology, Harbin, China; 2 College of Management and Economics, Tianjin University, Tianjin, China; 3 Southampton Clinical Trials Unit, University of Southampton, Southampton, United Kingdom

**Keywords:** digital technology, healthcare, information management, online health information, patient education

## 1 Introduction

Recent advances in digital technologies—such as online platforms, big data, live streaming, and artificial intelligence (AI)—are transforming how health and medical information is generated, shared, and managed, giving rise to new models of medical information management (1–3). Through digital health information systems, patients can access medical knowledge remotely, which has been shown to improve their wellbeing (4, 5). Within this broader shift, online health communities (OHCs) have become an increasingly important channel for patient care and education (6, 7). By offering fee-based online consultations, OHCs help patients overcome time and location constraints and obtain professional medical advice without the need for in-person visits (8, 9). Recent studies further show that the design of asynchronous interactions—such as communication depth, information intensity, and relationship duration—plays a key role in shaping patient satisfaction on these platforms, providing useful guidance for improving digital health information delivery (10, 11).

Nevertheless, existing research points to a persistent gap between the availability of digital health information and its effectiveness in supporting patient education (12). For example, studies of mobile health monitoring services show that affective factors—such as emotional attachment and affective attitudes—play a central role in sustained use, with user satisfaction and confirmation perceptions jointly shaping long-term engagement (13). Despite these insights, there remains limited guidance on how digital health information can be systematically designed and delivered to improve patient understanding and learning outcomes. This challenge is compounded by evidence that digital health innovations may unintentionally reinforce existing inequalities. High-quality online medical resources tend to be concentrated in economically developed regions, which can widen gaps in digital health literacy among vulnerable populations (14). Because effective health management requires patients to fully comprehend medical information, the ability to understand online health content is critical to the broader digital transformation of healthcare services (15). Yet much medical knowledge—particularly technical terminology—remains difficult for patients to interpret. Digital technologies offer promising ways to address this problem by enabling richer information types, formats, and presentation modes. These tools can enhance patients' understanding of medical knowledge and support more informed health-related decision making. One illustrative example is the emergence of online medical teams, which rely on multi-physician collaboration to deliver more comprehensive and authoritative advice (16). Recent evidence shows that such team-based digital models significantly increase patients' adoption of medical recommendations (17), highlighting a concrete pathway through which digital innovation can improve information comprehension and downstream behavioral outcomes.

This Research Topic aims to enhance patients' understanding of online health information by examining how digital health content is organized and presented, while highlighting the distinctive roles that mobile communication, big data, and AI play in contemporary healthcare. In this editorial, we outline the thematic focus of the Research Topic and highlight the key insights offered by the papers included in this Research Topic.

## 2 Papers in this research topic

This Research Topic brings together 13 articles that have successfully completed the standard peer-review process of Frontiers in Public Health. Although the studies differ in research focus, methodological approaches, and theoretical perspectives, they are united by a shared emphasis on digital information for patient education and address one or more of the predefined research themes. To provide a structured overview, we synthesize the contributions and organize them into six thematic areas: (1) medical and data-enabled technologies in information management for patient education; (2) the role of mobile health information and communication technologies (ICTs) in information sharing; (3) the provision of health information based on big data; (4) applications of AI in patient education; (5) the effectiveness of digital technologies in enhancing patient education outcomes; and (6) emerging digital innovations aimed at improving patients' understanding of health information.

Two studies address the theme of med-tech and data-enabled technologies in information management for patient education, examining how data-driven tools can enhance the organization and dissemination of health information. Huang, Guo et al. draw on motivation theory to examine how physicians' professional motivation shapes online knowledge sharing for patient education, with particular attention to the moderating roles of online experience and offline expertise. Using panel data from 11,839 physicians on a leading Chinese online health platform, the authors find that professional motivation positively influences both the quantity and quality of online knowledge sharing. Moreover, online experience strengthens this positive relationship, whereas offline expertise weakens the effect on sharing quantity. This study enriches the literature on motivation theory and online knowledge sharing and offers practical implications for physicians and platform managers. Liu et al. adopt a dual-factor model to investigate how discharged patients' individual characteristics affect their online health information-seeking behaviors, while considering the moderating role of living with children. Based on survey data from 292 discharged patients and analyzed using structural equation modeling, the results show that perceived stress encourages information-seeking behavior, whereas resistance to change and learned helplessness inhibit it. In addition, cohabitation with children moderates the negative effect of resistance to change. This study deepens our understanding of discharged patients' information-seeking behaviors and provides actionable insights for online health information providers.

Two articles focus on the role of mobile health ICTs in supporting information sharing for patient education. Using repeated cross-sectional surveys, Sakamoto et al. examine changes in parents' knowledge and concerns about pediatric fever between 2017 and 2024, as well as the association with the smartphone application “Oshiete! Doctor.” Their findings show that while parents' knowledge about fever improved substantially over time, anxiety related to potential brain damage and seizures increased. Moreover, the dissemination of the app alone was insufficient to counter broader societal anxiety. This study highlights the complex and evolving impact of mobile health applications on parental health literacy. Ma et al. investigate the dynamic evolution of users' continuance intention toward mHealth services by integrating the expectation–confirmation model with a latent growth modeling approach. Drawing on three waves of longitudinal survey data from 236 respondents, they find that continuance intention declines over time. Expectations accelerate this decline, whereas confirmation mitigates it, with the effect of expectation being more pronounced among users without chronic diseases. By extending the expectation–confirmation framework into a dynamic context, this study advances understanding of long-term mHealth service usage.

Two studies address the provision of health information driven by big data. Deng et al. conduct a scientometric and bibliometric analysis of medication literacy research published between 2003 and 2024, drawing on 1,968 articles from the Web of Science Core Collection and using CiteSpace and VOSviewer. Their big data analysis identifies three major research streams—medication literacy and adherence, the development of assessment tools, and psychosocial factors—and highlights key contributors, including the United States and Northwestern University. This study offers a comprehensive mapping of the field and helps clarify future research directions. Luo et al. analyze 331 atrial fibrillation–related scientific videos from YouTube, Bilibili, TikTok, and Douyin using big data techniques such as principal component analysis and normalization. The results show that videos produced by creators with medical backgrounds tend to attract greater popularity, whereas non-medical creators often provide higher-quality basic educational content. The study also points to limited content regulation across platforms. Together, these findings introduce novel approaches for large-sample, cross-platform analyses of digital health information.

Two articles examine the application of AI in patient education. Huang, Wang et al. assess the performance of five large language models—ChatGPT-4o, DeepSeek-V3, Doubao, Wenxin Yiyan 4.0 Turbo, and Qwen—in responding to 31 frequently asked ophthalmology consultation questions. Using a five-point Likert scale combined with textual analysis, the authors find that ChatGPT-4o and DeepSeek-V3 achieve the highest overall performance and are more suitable for lay users, whereas Doubao and Qwen are better aligned with the needs of medically trained audiences. This study offers practical guidance for the clinical deployment of large language models in ophthalmology. Bhattacharjee and Bhattacharya apply AI-driven nudge theory to improve hand hygiene compliance by introducing smart dispensers and wearable devices that deliver real-time visual and auditory reminders. Their findings show that these interventions can increase compliance by up to 30%, with additional gains when combined with gamification elements. This work highlights the potential of AI-enabled nudging technologies to support infection control and enhance patient education.

Three studies examine the effectiveness of digital technologies in patient education. Jiang et al. assess the quality of 200 Mandarin TikTok videos on diabetic retinopathy using the DISCERN and PEMAT-A/V instruments. Their analysis reveals substantial variation in content quality across uploader types, with videos produced by non-profit organizations scoring highest. Moreover, engagement metrics are strongly correlated with content quality, underscoring the importance of professional involvement and platform-level regulation in improving patient education outcomes. Impito and Azevedo evaluate the effectiveness of video-based health education (VbHE) in 33 least-developed African countries between 2020 and 2024, synthesizing evidence from 15 empirical studies. The findings indicate that VbHE can significantly enhance knowledge retention and health outcomes, particularly in areas such as cancer, HIV, and maternity care. At the same time, the authors highlight persistent challenges related to internet connectivity and cultural adaptation, emphasizing both the promise and the limitations of VbHE for underserved populations. Pavuloori et al. conduct a systematic review of 41 studies examining tools used to evaluate YouTube videos on surgical procedures. Their review shows that DISCERN, the Global Quality Score (GQS), and JAMA criteria are the most frequently applied assessment tools. However, they also find that approximately 88% of the videos fail to provide adequate patient education, while content from professional sources consistently offers higher educational value. This study calls for the development of standardized evaluation frameworks and improvements in the quality of online surgical education videos.

Two additional studies examine innovative digital technologies that enhance patients' understanding of health information. Zhou et al. conduct a cross-platform analysis of 271 pulmonary nodule–related videos published on YouTube, Bilibili, and TikTok, assessing both content quality and user engagement. Their findings indicate that long-form platforms such as YouTube and Bilibili tend to provide higher-quality medical information, whereas short-form platforms like TikTok generate greater user engagement. The study also shows that non-profit organizations and physicians are the primary content contributors. Based on these results, the authors offer practical recommendations for content creators, platform managers, and viewers. Wang et al. extend the theory of planned behavior to explore how sex-biased HPV vaccination information influences vaccination intentions among male college students. Using survey data from 240 respondents and structural equation modeling, they find that male-targeted information increases vaccination willingness by shaping attitudes, subjective norms, and perceived behavioral control. However, price sensitivity constrains the extent to which favorable attitudes translate into intention. This study provides valuable insights for designing gender-sensitive health communication strategies.

## 3 Conclusion

Research on digital information for patient education has grown rapidly, yet it remains fragmented across technological applications, information quality, and practical outcomes. This Research Topic addresses this gap by bringing together thirteen studies that examine key dimensions of digital patient education—ranging from med-tech, AI, and mobile health to big data–driven information provision and ethical considerations. Together, these articles offer both theoretical and empirical insights into how digital technologies shape patient education. The findings yield actionable implications for patients, clinicians, and platform designers, highlighting ways to improve digital health engagement and the delivery of medical information. At the same time, the Research Topic underscores several limitations, including regional specificity and the relative lack of evidence on long-term effects. These gaps point to promising avenues for future research, such as cross-platform comparisons, longitudinal analyses, and the integration of multiple digital technologies. Overall, this Research Topic provides a foundation for advancing digital patient education and encourages continued inquiry in this evolving field.
